# Saving and Empowering young lives in PAKistan (SEPAK): An Exploratory Cluster Randomized Controlled Trial (cRCT)

**DOI:** 10.1192/j.eurpsy.2023.703

**Published:** 2023-07-19

**Authors:** M. Panagioti, T. Kiran, S. Irshad, R. Sattar, A. Hodkinson, S. Tofique, N. Tyler, I. Angelakis, Z. Zadeh, A. Maqsood, S. Sultana, S. Dawood, M. Bhattia, A. Tamiduddin Nizami, H. A. Khan, I. B. Chaudhry, N. Husain, N. Chaudhry

**Affiliations:** ^1^Division of Population Health, Health Services Research & Primary Care, Institute for Health Policy and Organisation /Alliance Manchester Business School, Manchester, United Kingdom; ^2^Research, Pakistan Institute of Living and Learning, karachi, Pakistan; ^3^School of Psychology, University of South Wales, Wales, United Kingdom; ^4^Department of Behavioral Sciences, Fatima Jinnah Women University, Rawalpindi; ^5^Department of Applied Psychology, Bahauddin Zakariya University, Multan; ^6^Center for Clinical Psychology, University of Punjab, Lahore; ^7^Department of Psychiatry, Peoples University of Medical and Health Sciences for Women, Nawabshah; ^8^Department of Medicine, Institute of Psychiatry, Benazir Bhutto Hospital, Rawalpindi; ^9^Baloshistan Institute of Psychiatry & Behavioral Sciences, Bolan Medical College, Quetta; ^10^Division of Psychology and Mental Health, University of Manchester, Manchester, Pakistan

## Abstract

**Introduction:**

Suicide is a leading cause of death among young people and most deaths by suicide occur in low and middle-income countries. School is the best place where we can identify and respond to youth suicide risk. School-based interventions for suicide prevention in young people have been successful across US, Europe and Australia, but require adaptations to be acceptable and feasible in Pakistan.

**Objectives:**

To develop and test culturally adapted preventative interventions for suicidal behaviours among pupils in secondary schools in Pakistan. The qualitative component aimed at exploring the views of students, parents, teachers and general practitioners on cultural adaptation, experience of participation, areas of improvement and suggestions for scale-up of the school-based suicide prevention program (SEPAK).

**Methods:**

A clustered randomised controlled trial. The four culturally modified interventions 1) Linking Education and Awareness of Depression and Suicide Awareness (LEADS) Training for pupils (students=260) 2) the Question, Persuade, and Refer (QPR) for teachers (students=203) 3) QPR for parents (students=445); 4) Screening by Professionals (Profscreen) (students=260) were compared against control intervention (educational posters) (students=227). Structured questionnaires were administered at baseline and 1-month post-intervention to assess suicidal behaviours, psychological well-being and quality of life. A total of 8 focus groups (FGs) were conducted at pre and post intervention stage with each stakeholders.

**Results:**

Patient and public involvement and Engagement (PPIE) was strongly embedded in the project to ensure meaningful benefits for participants. A total of 40 schools were recruited from 8 cities across Pakistan. A total of 243 students attended LEADS intervention, 92 teachers and 304 parents completed QPR training, and 9 general practitioners were trained in ProfScreen. The retention rate at follow-up was 99% that shows feasibility of delivering intervention package in Pakistan. All participants marked SEPAK as effective in identifying risk of and preventing self-harm and suicide in young people and in improving pathways to treatment. Interventions were perceived as helpful in improving knowledge about mental health, impact of mental health difficulties on functioning, reducing stigma, equipping stakeholders to identify and signpost at-risk people. Improvement in clinical and teaching practice as well as understanding others behaviors were also reported.

**Conclusions:**

This study suggest feasibility of integrating a suicide prevention program in existing educational system and highlights positive role of creating awareness about suicide in youth, introduction of school-based mental health programs, parental counseling and strengthening of the health system by training general practitioners in early identification of suicide risk and promoting suicide prevention strategies

**Disclosure of Interest:**

None Declared

